# Supplementary material for: High prevalence and extended deletions in Plasmodium falciparum hrp2/3 genomic loci in Ethiopia
Source: PLoS One. 2020 Nov 5;15(11):e0241807. doi: 10.1371/journal.pone.0241807 (PMC7644029; doi:10.1371/journal.pone.0241807)
Supplement: S1 File — (PDF) [file pone.0241807.s001.pdf]

Additional file 1. Summary of *pfhrp*2, *pfhrp*3 amplification and their respective flanking genes in *P. falciparum* samples collected in Ethiopia.

No.of samples	PF3D7_0831700	HRP2	PF3D7_0831900	PF3D7_1372100	HRP3	PF3D7_1372400
20	+	-	+	-	-	+
16	+	-	+	-	-	-
8	+	-	-	-	-	+
3	+	-	-	-	-	-
2	-	-	+	-	-	-
1	+	-	+	+	-	+
50						
