# Supplementary material for: High prevalence and extended deletions in Plasmodium falciparum hrp2/3 genomic loci in Ethiopia
Source: PLoS One. 2020 Nov 5;15(11):e0241807. doi: 10.1371/journal.pone.0241807 (PMC7644029; doi:10.1371/journal.pone.0241807)
Supplement: S2 File — (DOC) [file pone.0241807.s002.doc]

PCR protocol used for amplification of *Plasmodium* DNA

.6µl of water

.10µl of RedTaq master mix

.* Composition of VWR Taq 2x Master Mix

• Tris-HCl pH 8.5, (NH4)2S04, 4.0 mM MgCl2, 0.2 % Tween® 20

• 0.4 mM of each dNTP

• 0.2 units/μl VWR Taq polymerase

• Stabilizer

.1µl of each primer (conc 10µM)

.2µl of DNA-preparation (extracted from filter paper, concentration not measured)

Standard agarose gel electrophoresis with TBE buffer including GelRed for detection. Gels read in BioRad Gel Viewer.
