# Supplementary material for: High prevalence and extended deletions in Plasmodium falciparum hrp2/3 genomic loci in Ethiopia
Source: PLoS One. 2020 Nov 5;15(11):e0241807. doi: 10.1371/journal.pone.0241807 (PMC7644029; doi:10.1371/journal.pone.0241807)

**Supplementary documents 2**: Pfhrp2/3 gene amplification for Tanzanian samples (both positive and negative) and Ethiopian samples (all negatives).

a). Tanzanian samples tested positives for hrp2/3 genes


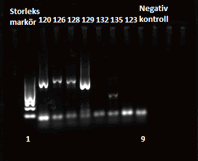


b). Ethiopian samples tested negatives for hrp2/3 genes (please note that no positive control is included)


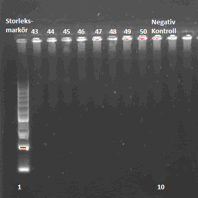

Supplement: S3 File — a). Tanzanian samples tested positives for hrp2/3 genes. b). Ethiopian samples tested negatives for hrp2/3 genes (please note that no positive control is included). (DOC) [file pone.0241807.s003.doc]
