# Supplementary material for: High prevalence and extended deletions in Plasmodium falciparum hrp2/3 genomic loci in Ethiopia
Source: PLoS One. 2020 Nov 5;15(11):e0241807. doi: 10.1371/journal.pone.0241807 (PMC7644029; doi:10.1371/journal.pone.0241807)
Supplement: Attachment — Submitted filename: Response to Reviewers, PLOS_LG.doc [file pone.0241807.s004.doc]

17 September 2020

Academic Editor:

**Editor’s comment:** 1. Please ensure that your manuscript meets PLOS ONE's style requirements, including those for file naming. The PLOS ONE style templates can be found at

<https://journals.plos.org/plosone/s/file?id=wjVg/PLOSOne_formatting_sample_main_body.pdf> and

<https://journals.plos.org/plosone/s/file?id=ba62/PLOSOne_formatting_sample_title_authors_affiliations.pdf>

**Author response:** Yes, we prepared our manuscript as per the PLOS ONE journal style requirements.

**Editor’s comment:** 2. Thank you for including the following funding information within the acknowledgements section of your manuscript; "This work was supported through the DELTAS Africa Initiative [DELGEME grant 107740/Z/15/Z]. The DELTAS Africa Initiative is an independent funding scheme of the African Academy of Sciences (AAS)’s Alliance for Accelerating Excellence in Science in Africa (AESA) and supported by the New Partnership for Africa’s Development Planning and Coordinating Agency (NEPAD Agency) with funding from the Wellcome Trust [DELGEME grant 107740/Z/15/Z] and the UK government. "

We note that you have provided funding information that is not currently declared in your Funding Statement. However, funding information should not appear in the Acknowledgments section or other areas of your manuscript. We will only publish funding information present in the Funding Statement section of the online submission form.

Please remove any funding-related text from the manuscript and let us know how you would like to update your Funding Statement. Currently, your Funding Statement reads as follows:

"No, the funders had no role in study design, data collection and analysis, decision to publish, or preparation of the manuscript."

**Author response:** Funding information is removed from the manuscript as suggested. Moreover, we updated Funding Statements and now it reads “Yes, the funders had no role in study design, data collection and analysis, decision to publish, or preparation of the manuscript."

**Editor’s comment**: 3. Thank you for including your competing interests statement;

"No, I have read the journal's policy and the authors of this manuscript have the following competing interests"

Please complete your Competing Interests on the online submission form to state any Competing Interests. If you have no competing interests, please state "The authors have declared that no competing interests exist.", as detailed online in our guide for authors at http://journals.plos.org/plosone/s/submit-now

 This information should be included in your cover letter; we will change the online submission form on your behalf.

**Author response:** Thanks, now the competing interest statement reads ‘The authors have declared that no competing interests exist’. We urged you to correct this in the cover letter on our behalf.

**Reviewer #1**: In this study, the authors determined the deletions of pfhrp2 and pfhrp3, as well as of genes upstream and downstream of these two genes, by PCR analysis of samples from patients infected with Plasmodium falciparum in Ethiopia. The authors show that in a substantial number of samples, the parasite lacks both pfhrp2 and pfhrp3, which may lead to pseudo-negative results when using HRP2-based RDTs for testing individuals suspected to have malaria in Ethiopia. I think some modifications and additional explanations are required.

(1) Abstract (Conclusion, line 5 from the bottom): Because the term “fixed” might have the same meaning as “repaired,” I was confused when I read the manuscript for the first time. In this paper, did you mean “found?” I think it would be better to rephrase the sentence.

**Author response:** Sorry for the inconvenience, we have now replaced the word ‘fixed’ by ‘found’ across the text to avoid the confusion.


(2) Introduction (Paragraph 3, line 9): Please replace “pfhr2/3loci” with “pfhrp2/3 loci.”

**Author response:** Thanks, we have corrected “pfhr2/3loci” with “pfhrp2/3 loci as commented.

(3) Introduction (Paragraph 4, lines 1 to 6 from the bottom): The sentences from “As per” to “of this mutation” should be moved to the Discussion section.

**Author response:** Thanks we moved these sentences to the discussion section.

(4) Methods: I think the information about the pseudo-positive rates of the HRP2-based RDTs is important. Do you have results of the RDTs for samples from the 50 patients infected with hrp2- hrp3- parasite?

**Author response:** Unfortunately, we collected *P.falciparum* positive samples based on results of microscope slide readings and we don’t have RDT results for 50 of the samples. This is indeed stated as the limitation of the study.

(5) Results (p. 14, line 12): The term “fixed” should be replaced with an appropriate term.

**Author response:** Sorry for the inconvenience, we have now replaced the word ‘fixed’ by ‘found’ across the text to avoid the confusion.

(6) Results (p. 14, line 4): You have annotated “Table 3;” however, important results and/or information are included in Additional file 1. You should annotate Additional file 1 as Table 3.

**Author response:** Thanks, now additional file1 is annotated as Table 3 as suggested.

(7) Table 3: I cannot understand what have you listed in Table 3. Why did you list the five samples?

**Author response:** Indeed, table 3 bears the summary of deletions observed in pfhrp2/3 genes including the flanking regions in 50 of the samples, but does not bear list of 5 samples. The list of five samples is rather shown on Table 4 where AT repeats were seen.

**Reviewer #2:**

This manuscript reports the results of a small study assaying the prevalence of Pfhrp2 and 3 deletions in 50 samples from a single collection point in Ethiopia collected between September and November 2015. The authors report that all parasites characterised had deletions of both genes, presumably rendering them non-detectable by the commonly used PfHRP2 based RDTs used in this area. The methodology is appropriate, and the results of interest. I have several points that the authors may consider for revision:

As there are no page numbers on the document, I have numbered them myself, with Page 1 of the PDF being the abstract…

General

**Reviewer’s comment**: The manuscript needs a thorough proofreading to correct numerous typological, spelling and grammatical errors.

**Author’s response:** Thanks, we have tried our best to undertake English language proofreading, correct typological errors, grammars and spelling to the best of our understanding.

**Reviewer’s comment**: Please could data on the malaria endemicity of the region from which samples were collected be given? Is this a high transmission region for P. falciparum?

**Author’s response:** We appreciate the comments; we have now included the species of malaria circulating in the study site in the MM section.The study site exhibits high malaria transmission with both *P.falciparum* and *P.vivax* species co-transmit in the study site.

**Reviewer’s comment**: Samples are rather few, and are from a very specific region of Ethiopia. It is very difficult to ascertain whether the results here are representative of a the wider Ethiopian P. falciparum population or simply constitute a small local population. There is no description of the diagnostics commonly used in this region of Ethiopia – as the patients here were diagnosed by microscopy, is it right to assume this is the standard protocol? In which case, presumably, RDTs are not used here. In which case, the authors need to offer some hypothesis of why HRP2 deleted parasites dominate in a region where there should have not undergone selection….

**Author’s response:** Yes, the samples are quite low in numbers to arrive at a definitive conclusion regarding pfhrp2/3 gene deletions in the Ethiopian *P.falciparum* populations, however, given the Adama malaria diagnostic center is one of the oldest laboratories for malaria in Ethiopia, it is preferred over any other diagnostic laboratories in the area as far as malaria diagnosis is concerned. This is because WHO certified laboratory technicians are working for this center. Moreover, the surrounding people usually prefer this center to undertake diagnosis for malaria. As the center exclusively performs malaria diagnosis, febrile patients from the town and the surrounding rural areas enjoy visiting this center to rule out malaria infection. In the surrounding rural areas, for instance, pfhrp2/3-based RDTs are massively used for diagnosis. Even at Adama malaria diagnosis center, RDTs are used when electricity is unavailable. In one way or another, the P. falciparum isolates circulating in the town and the surrounding rural areas are exposed to high levels of diagnosis pressure by pfhrp2/3-based RDTs leading to ‘diagnostic selection’. Although Adama malaria diagnostic center does microscopic diagnosis (and rarely use RDTs) most of the time, the patients coming to this center might have been exposed to pfhrp2/3-based RDTs for more than fifteen years in Ethiopia and may carry parasites with deleted pfhrp2/3 genes as part of the survival mechanism to remain undetected and untreated.

**Reviewer’s comment**: There is a general lack of essential information in the introduction; it would be beneficial for the reader to know not only the malariometrics of the study area (entomological inoculation rates, malaria parasite species present, parasite prevalence etc), but also the extent to which RDTs are used for diagnosis, and when they were introduced.

**Author’s response:**  We appreciate the reviewer comment. We have now included a paragraph to describe the extent to which RDTs are used for diagnosis, and when they were introduced in the introduction section as commented.

Introduction

**Reviewer’s comment**: Page 2, sentence 1: please use percentages (60%) instead of “in 0.60 of cases”.
**Author’s response**: Thanks, corrected 60%.

**Reviewer’s comment**: Page 2, sentence 3. 40% reduction since when?

**Author’s response:** 40% reduction in the incidence of clinical disease between 2000 and 2015 and this is clearly indicated in the edited version of the manuscript.

**Reviewer’s comment**: Page 2, paragraph 3. The authors contend that PfHRP2 deletions have not been extensively surveyed in Africa. There are, in fact, very many reports detailing such surveys.

**Author’s response:** yes, there are several studies in Africa in connection to pfhrp2/3 gene deletions, however, such info is lacking in Ethiopia. Thus, it should read in Ethiopia.

Methods:

**Reviewer’s comment**: Page 4. Please at least give the primer names and conditions for the PCR rather than referring to previous publications

**Author’s response:** Thanks, now primers name and the PCR conditions are included in the manuscript.

**Reviewer’s comment**: Page 6. The positive control samples (with the Pfhrp2 genes intact) come from Tanzania. Is it possible that the strains circulating in Ethiopia have different sequences at the primer binding sights in hrp2/3 genes, rendering the PCR ineffective? Why wasn’t an Ethiopian PfHRP2 intact P. falciparum used as a positive control?

**Author’s response:** Indeed, we analyzed *pfhrp*2/3 gene deletions in isolates collected from Tanzania and Ethiopia although we didn’t include results obtained from Tanzanian isolates in our analysis. While the Ethiopia isolates exhibited extensive deletions, the deletion was low among the Tanzanian isolates. This confirms that the primers were working well as far as the detection of pfhrp2/3 gene deletions is concerned. By the positive control samples from Tanzania, we mean that our primers and PCR conditions work very well in samples from Tanzania. Although the circulating P. falciparum strains in Ethiopia and Tanzania *per se* may be distinct, the primer binding sites in *hrp2/3* genes cannot be different. Unfortunate, we didn’t include intact Ethiopian PfHRP2 P. falciparum as a positive control.

Discussion
**Reviewer’s comment**: Page 11 paragraph 1 – the authors find three deletion variants according to the size of the deletion. This does not fit with proposal of clonal expansion of one mutant.

**Author’s response:** Yes, authors find three deletion variants as opposed to the observed clonal expansion of one mutant observed in another study conducted in the Amazon region of Peru, as cited in the text [8].

6. PLOS authors have the option to publish the peer review history of their article ([what does this mean?](https://journals.plos.org/plosone/s/editorial-and-peer-review-process#loc-peer-review-history)). If published, this will include your full peer review and any attached files.


**Reviewer’s comment**: If you choose “no”, your identity will remain anonymous but your review may still be made public.


**Do you want your identity to be public for this peer review?** For information about this choice, including consent withdrawal, please see our [Privacy Policy](https://www.plos.org/privacy-policy).

Reviewer #1: No

Reviewer #2: **Yes:** Richard Culleton

[NOTE: If reviewer comments were submitted as an attachment file, they will be attached to this email and accessible via the submission site. Please log into your account, locate the manuscript record, and check for the action link "View Attachments". If this link does not appear, there are no attachment files.]

While revising your submission, please upload your figure files to the Preflight Analysis and Conversion Engine (PACE) digital diagnostic tool, <https://pacev2.apexcovantage.com/>. PACE helps ensure that figures meet PLOS requirements. To use PACE, you must first register as a user. Registration is free. Then, login and navigate to the UPLOAD tab, where you will find detailed instructions on how to use the tool. If you encounter any issues or have any questions when using PACE, please email PLOS at [figures@plos.org](mailto:figures@plos.org). Please note that Supporting Information files do not need this step.
